# Peptidoglycan Recognition Peptide 2 Aggravates Weight Loss in a Murine Model of Chemotherapy-Induced Gastrointestinal Toxicity

**DOI:** 10.3389/fonc.2021.635005

**Published:** 2021-03-23

**Authors:** Ann-Sophie Bech, Anders Bathum Nexoe, Magdalena Dubik, Jesper Bonnet Moeller, Grith Lykke Soerensen, Uffe Holmskov, Gunvor Iben Madsen, Steffen Husby, Mathias Rathe

**Affiliations:** ^1^Hans Christian Andersen Children's Hospital, Odense University Hospital, Odense, Denmark; ^2^Department of Cancer and Inflammation Research, Department of Molecular Medicine, University of Southern Denmark, Odense, Denmark; ^3^Department of Medical Gastroenterology, Odense University Hospital, Odense, Denmark; ^4^Danish Institute for Advanced Study (D-IAS), University of Southern Denmark, Odense, Denmark; ^5^Department of Pathology, Odense University Hospital, Odense, Denmark; ^6^Department of Clinical Research, University of Southern Denmark, Odense, Denmark

**Keywords:** peptidoglycan recognition peptide 2, gastrointestinal mucositis, mice, inflammation, chemotherapy

## Abstract

**Introduction:** Chemotherapy-induced gastrointestinal toxicity (CIGT) is a frequent, severe and dose-limiting side effect. Few treatments have proven effective for CIGT. CIGT is characterized by activation of the nuclear factor kappa B pathway which, leads to upregulation of proinflammatory cytokines. The innate immune protein peptidoglycan recognition peptide 2 (PGLYRP2) binds to and hydrolyzes microbial peptidoglycan. Expression of *PGLYRP2* is upregulated in the intestine of chemotherapy-treated piglets. In this experimental study, we investigated the role of *Pglyrp2* in the development and severity of murine CIGT.

**Methods:** *Pglyrp2* wildtype and *Pglyrp2* knockout mice received intraperitoneal injections of chemotherapy (Doxorubicin 20 mg/kg) to induce CIGT. Weight was monitored daily, and animals were euthanized after 2 or 7 days. Expression of proinflammatory cytokines in the jejunum was measured by quantitative real-time polymerase-chain reaction and enzyme-linked immunosorbent assay. Villus height, crypt depth, and histologic inflammation were evaluated on haematoxylin and eosin stained tissue specimens.

**Results:** Chemotherapeutic treatment induced weight loss (*p* < 0.05), shortening of the small intestine (*p* < 0.05), elongation of villus height (*p* < 0.05), increased crypt depth (*p* < 0.05), and led to elevated mRNA levels of *II1β* (*p* < 0.05), *II6* (*p* < 0.05), and *Tnf* (*p* < 0.001) at day 2. Protein levels of IL1β, IL6, and TNFα did not change after exposure to chemotherapy. Doxorubicin treated wildtype mice had a more pronounced weight loss compared to knockout mice from day 3 to day 7 (D3-D6: *p* < 0.05 and D7: *p* < 0.01). No other phenotypic differences were detected.

**Conclusion:** *Pglyrp2* aggravates chemotherapy-induced weight loss but does not induce a specific pattern of inflammation and morphological changes in the small intestine.

## Introduction

Chemotherapy-induced gastrointestinal toxicity (CIGT) is a frequent and painful side effect of chemotherapeutic treatment characterized by mucosal ulcerations in the digestive tract. It affects 40–100% of cancer patients treated with chemotherapy (1, 2). Clinical signs of CIGT are oral and abdominal pain, nausea, vomiting and diarrhea (3, 4). CIGT increases the risk of bleeding, malnutrition (5, 6), and potentially life-threatening infections (7, 8). More than 63% of cancer patients who undergo chemotherapy experience unintentional weight loss, and weight loss is associated with symptoms of CIGT (4). Weight loss is incorporated in several grading systems of gastrointestinal mucositis (1). Nutritional status is important as a healthy nutritional status may improve tolerance to treatment and survival (9, 10). Thus, the effects of gastrointestinal toxicity on nutritional status may have implications for treatment outcomes (11–14).

Few treatments have proven effective for CIGT. Current therapy is based on basic oral hygiene, appropriate analgesia and management of infections (7). Since there is no effective treatment for CIGT it is often necessary to reduce or delay the chemotherapeutic treatment (15).

The exposure of the gastrointestinal mucosa to a cytotoxic agent generates reactive oxygen species, activation of transcription factors (16, 17) and increased expression of a number of proinflammatory cytokines. Tumor necrosis factor alpha (TNF), interleukin 1β (IL1β) and interleukin 6 (IL6) are considered central for the development of CIGT (18).

Peptidoglycan recognition peptide 2 (PGLYRP2) is a pattern recognition receptor in the innate immune system (19) that binds, hydrolyzes, and neutralizes proinflammatory bacterial peptidoglycan (20, 21). *Pglyrp2* have shown both protective anti-inflammatory (22, 23) and disease-inducing proinflammatory properties in murine disease models (24).

We have previously shown that *PGLYRP2* is upregulated in the porcine jejunum during chemotherapeutic treatment, which indicates that *PGLYRP2* could be involved in protection of mucosal surfaces (25).

In the present study we investigate if *Pglyrp2* deficiency affects CIGT in mice. Weight loss was monitored daily. Expression of inflammatory cytokines and severity of structural damage to the gastrointestinal mucosa were evaluated 2 and 7 days after induction of CIGT in *Pglyrp2* wildtype (WT) and knockout (KO) mice.

## Materials and Methods

### Ethics, Permissions and Humane Endpoints

All experiments were performed with prior approval from The Danish Animal Experiments Inspectorate (Approval no. 2017-15-0201-01385 and 2017-15-0202-00110). Animals were assessed daily by experienced animal technicians and euthanized by cervical dislocation if they showed sign of pain or poor well-being or if weight loss exceeded 20%.

### Study Design and Setting

The study was an experimental animal study performed from June to September 2019 in the Biomedical Laboratory at the University of Southern Denmark, Odense, Denmark. 30 *Pglyrp2* KO and 33 *Pglyrp2* WT mice were included in the study. Mucositis induction was achieved by doxorubicin (Doxo) treatment as described earlier (26). At day−1 baseline weight was measured. At day 0 Doxo 20 mg/kg or saline (NaCl) was administered by intraperitoneal (IP) injections. Animals were randomized to either the control group receiving NaCl (WT-NaCl, *n* = 5 and KO-NaCl, *n* = 5) or to induction of mucositis with Doxo. Animals receiving Doxo were either euthanized day 2 (WT-Doxo 2, *n* = 10 and KO-Doxo 2, *n* = 11) or day 7 (WT-Doxo 7, *n* = 18 and KO-Doxo 7, *n* = 14, see Supplementary Figure 1).

### Animals; Breeding and Housing

C57BL/6J Jax *Pglyrp2*–/– and C57BL/6NTac *Pglyrp2*+/+ mice were interbred in-house and the offspring was used for this study. Female mice 10–12 weeks of age from N1F2 generation were used in the experiment. Littermates were co-housed until 1 week prior to inclusion, at this time the mice were transferred from group housing to individual cages to acclimatize. The mice were housed in single disposable cages provided with water and food *ad libitum* at 20–24°C, 55% humidity and a 12-h light/dark cycle.

### Genotyping

Genotyping was performed on ear punch biopsies before inclusion and on tail biopsies after euthanasia as previously described (27) using primers as shown in Supplementary Table 1.

### Induction of Mucositis

Doxo (Doxorubicin hydrochloride 2 mg/ml, Accord, North Harrow, Great Britain) was diluted 1:1 in sterile saline (NaCl 9 mg/ml, B. Braun, Melsungen, Germany) prior to administration to a working solution of 1 mg/ml. The dose was administered as two IP injections.

### Euthanasia and Data Collection

Weight was measured daily between 8:00 a.m. and 11:00 a.m. Doxo-treated animals experiencing a weight loss <5% were excluded from the study due to presumed injection error (28, 29). The mice were anesthetized by IP injection of ketamine 100 mg/kg (Ketaminol® Vet, MSD Animal Health, Boxmeer, The Netherlands) and xylazine 10 mg/kg (Rompun® vet, Bayer Animal Health GmbH, Leverkusen, Germany) and subsequently exsanguinated by collecting a blood sample by cardiac puncture. Animals were perfused with phosphate-buffered saline and a tail biopsy was collected. The small intestine was removed from the pyloric sphincter to the ileocecal junction and divided into three equally sized pieces representing duodenum, jejunum and ileum for use in histopathological analysis, quantitative real time polymerase chain reaction (qRT-PCR), and enzyme-linked immunosorbent assay (ELISA). The length of these segments was measured, and the segments were flushed with phosphate-buffered saline. Samples for histopathological analysis were fixated in 4% formaldehyde (VWR Chemicals, Leuven, Belgium) for 48 h, transferred to phosphate-buffered saline with 0.05% NaN_3_, embedded in paraffin, mounted on glass slides, and stained with haematoxylin and eosin (HE) at the Department of Pathology, Odense University Hospital, Odense, Denmark.

### Data Analysis

#### Histopathology

Villus height and crypt depth were measured on electronic copies of HE-stained tissue using the NDP.view2 software (Hamamatsu Photonics, Hamamatsu City, Japan). For each mouse five to ten villus-crypt units were measured in the duodenum, jejunum, and ileum, respectively. Villus height was measured from the villus-crypt junction to the villus tip in villi with a single layer of epithelial cells cut through the nuclei all the way around the villus. Crypt depth was measured from the base of the crypt to the villus-crypt junction in crypts with open lumens and a continuous cell column on each side.

A blinded pathologist (GM) graded morphological changes in HE-stained jejunal samples from 0 to 5 according to the following previously described scaling system (30): 0, Normal mucosal villus; 1, Development of subepithelial Gruenhagen's space, usually at the apex of villus; often with capillary congestion; 2, Extension of the subepithelial space with moderate lifting of epithelial layer from the lamina propria; 3, Massive epithelial lifting down the sides of villi. A few tips may be denuded; 4, Denuded villi with lamina propria and dilated capillaries exposed. Increased cellularity of lamina propria may be noted; 5, Digestion and disintegration of lamina propria; hemorrhage and ulceration.

#### Quantitative Real-Time Polymerase Chain Reaction

The expression of *Pglyrp2, Il1*β, *Il6*, and *Tnf* were measured in jejunal samples. RNA was isolated from jejunal tissue samples with 1 ml TRIzol^TM^ Reagent (Invitrogen^TM^, Carlsbad, CA, USA) according to the manufacturer's instruction. The concentration and purity of RNA was quantified by spectrophotometry using the NanoDrop^TM^ One (Thermo Scientific, Wilmington, DE, USA). All RNA samples had A260/A280 ratio >1.9 suggesting adequate purity. 1 μg of RNA was used for cDNA synthesis. Reverse transcription of total RNA was performed using High-Capacity cDNA Reverse Transcription Kit (Thermo Fisher Scientific, Vilnius, Lithuania) according to the manufacturer's protocol. qRT-PCR analyses were performed using the TaqMan^TM^ Universal Mastermix II, no UNG (Applied Biosystems, Foster City, CA, USA) using TaqMan Gene Expression Assays (Applied Biosystems, Pleasanton, CA, USA) as shown in Supplementary Table 2. The levels of gene expression were quantified using StepOnePlus^TM^ Real-Time PCR-System (Applied Biosystems, Foster City, CA, USA) and normalized as n-fold difference to mouse *Hprt*.

#### Enzyme-Linked Immunosorbent Assay

The protein levels of PGLYRP2, IL1β, IL6, and TNFα were measured in jejunal samples. One centimeter jejunal tissue specimens (weight, 350–500 mg, stored at −80°C) were homogenized using a Precellys 24 homogenizer in 800 μl PBS containing EDTA-free protease inhibitor (Roche Applied Science, Penzberg, Germany). Homogenized tissue supernatants were standardized to 1 mg/ml after protein concentration measurements using the DC protein assay according to the manufacturer's recommendations (Bio-Rad Laboratories, Hercules, CA, USA). Measurements of IL1β, IL6, and TNFα by ELISA were performed using DuoSet® ELISA according to the manufacturer's instructions (R&D Systems, Inc., Minneapolis, MN, USA). Measured cytokine concentrations were standardized to the initial weight of sample. We evaluated commercially available ELISA kits for PGLYRP2 (Amsbio, Abingdon, U.K.), however they turned out not to be specific for PGLYRP2 and thus cannot be trusted. Data is not shown. For details on ELISA kits see Supplementary Table 3.

#### Statistical Analysis

Normal distribution of data was determined using D'Agostino & Pearson test. Difference in weight at baseline was analyzed by one-way ANOVA followed by Tukey's multiple comparison test. Difference in weight, small intestinal length, villus height, crypt depth, villus crypt ratio, histological score, qRT-PCR data and ELISA data between groups were analyzed by two-way ANOVA followed by either Tukey's multiple comparison test or Holm-Sidak multiple comparison test as noted in figure legends for data following a Gaussian distribution. Kruskal–Wallis test with Dunn's multiple comparison test was used to analyze data not following a Gaussian distribution. All data are presented as mean values ± standard error of the mean (SEM). A *p* < 0.05 was considered statistically significant. All statistical analyses were performed using GraphPad Prism version 8.1.1 (GraphPad Software, San Diego, CA, USA).

## Results

### Animals

A total of 63 mice were included in the experiments. Three mice, two WT-Doxo 7 and one KO-Doxo 7, were excluded due to weight loss >20% following Doxo treatment and two KO-Doxo 7 mice were excluded due to weight loss <5% following Doxo treatment due to assumed injection error (28, 29). One mouse from KO-Doxo 2 was excluded as it was heterozygote after control genotyping leaving a total sample size of 57 mice.

### Weight Loss

**Figure 1:**
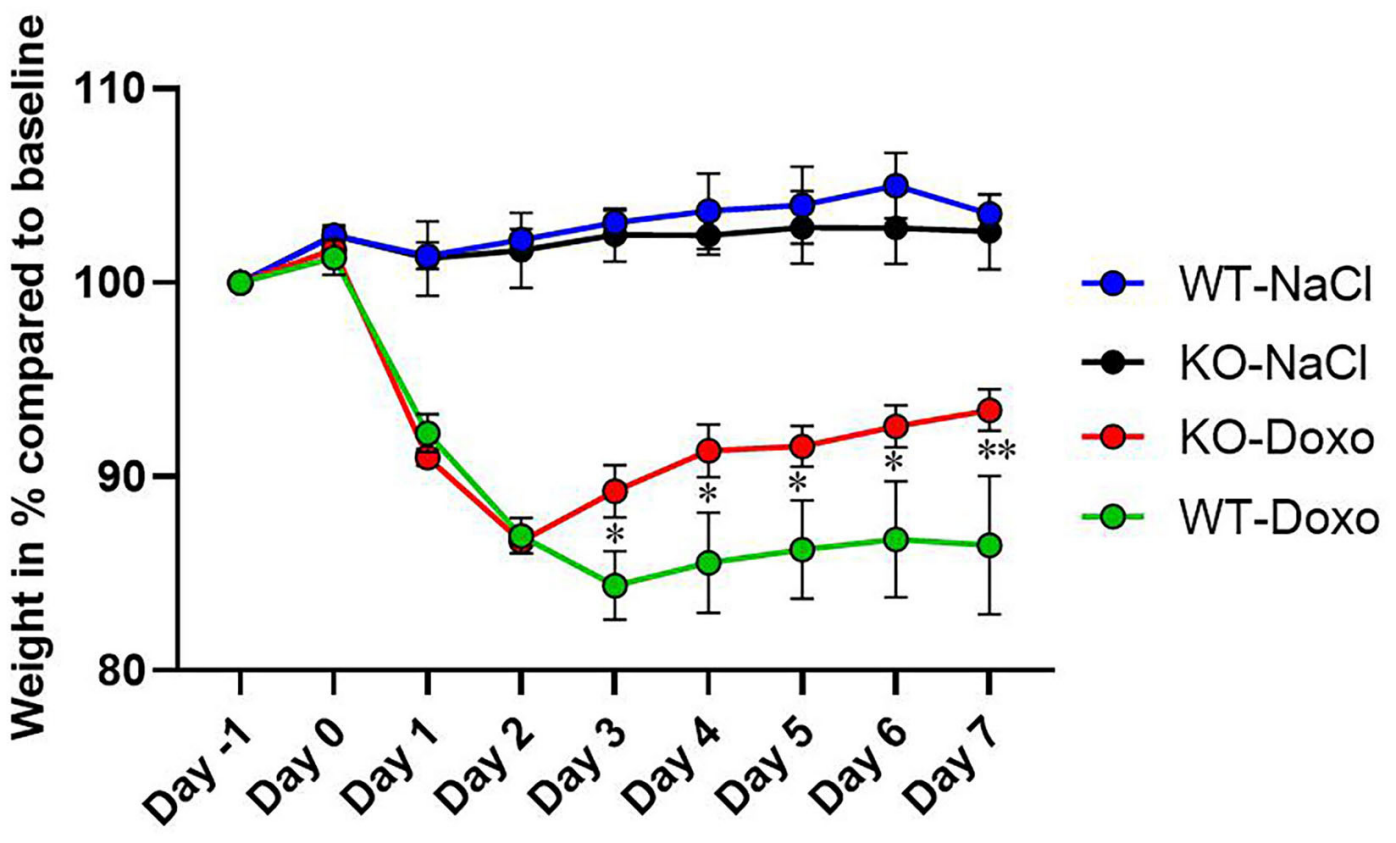
Weight loss. Change of weight in % each day as compared to baseline weight (Day−1) in wildtype (WT) and knockout (KO) mice. WT-NaCl, *n* = 5. KO-NaCl, *n* = 5. WT-Doxo 2, *n* = 10. KO-Doxo 2, *n* = 10. WT-Doxo 7, *n* = 16. KO-Doxo 7, *n* = 11. Data is presented as means ± standard error of the mean. Two-way ANOVA followed by Tukey multiple comparison test was used to analyze results. *p < 0.05 and **p < 0.01.

At day 0, there was no difference in mean weight between the experimental groups confirming that mice were allocated to groups independent of their weight at start of the experiment. At each day, from day 1 to day 7, there was a significant weight reduction in mice receiving Doxo compared to mice receiving NaCl (*p* ≤ 0.05) (Figure 1). From day 3 to day 7 WT-Doxo had a significant greater weight loss than KO-Doxo (D3-D6: *p* < 0.05 and D7: *p* < 0.01). Maximum weight loss was reached at day 2 for KO-Doxo and at day 3 for WT-Doxo. KO-Doxo had a smaller total weight loss and faster weight gain as compared to WT-Doxo (Figure 1).

### Length of the Small Intestine

**Figure 2:**
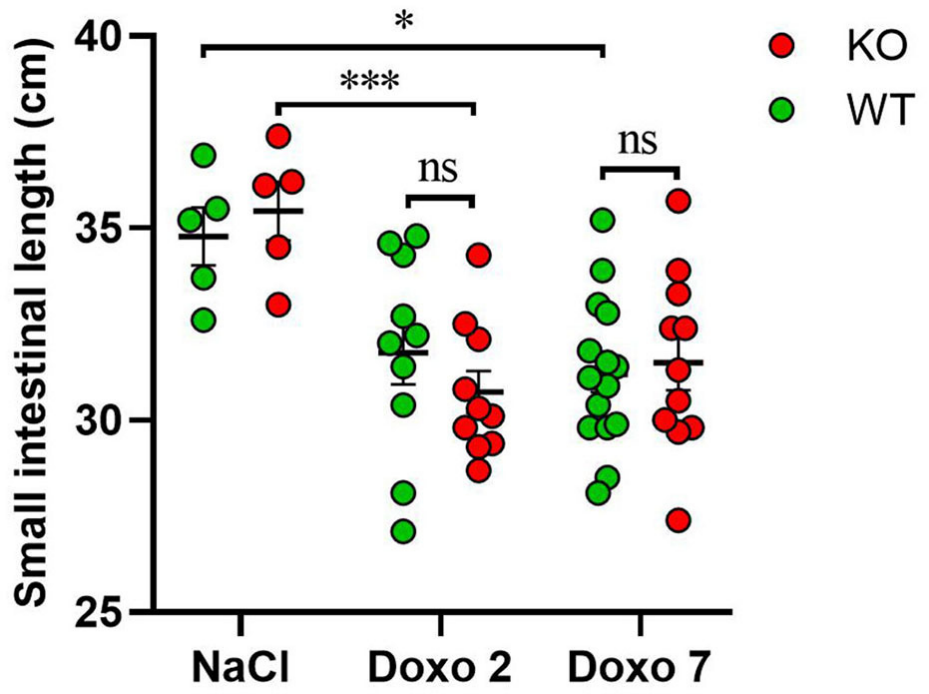
Small intestinal length in cm in wildtype (WT) and knockout (KO) mice 7 days after IP injection with saline (NaCl) or 2 or 7 days after IP injection with doxorubicin (Doxo). WT-NaCl, *n* = 5. KO-NaCl, *n* = 5. WT-Doxo 2, *n* = 10. KO-Doxo 2, *n* = 10. WT-Doxo 7, *n* = 16. KO-Doxo 7, *n* = 11. Data is presented as means ± standard error of the mean. Two-way ANOVA followed by Holm-Sidak multiple comparison test was used to analyze results. *p < 0.05, ***p < 0.001, and ns, no significance.

The length of the small intestine was significantly shortened in KO-Doxo 2 mice compared to KO-NaCl (*p* < 0.001) and in WT-Doxo 7 compared to WT-NaCl mice (*p* < 0.05) (Figure 2). There was no difference in small intestinal length after chemotherapeutic treatment that could be attributed to the genotype.

### Villus Height, Crypt Depth, and Villus Crypt Ratio

**Figure 3:**
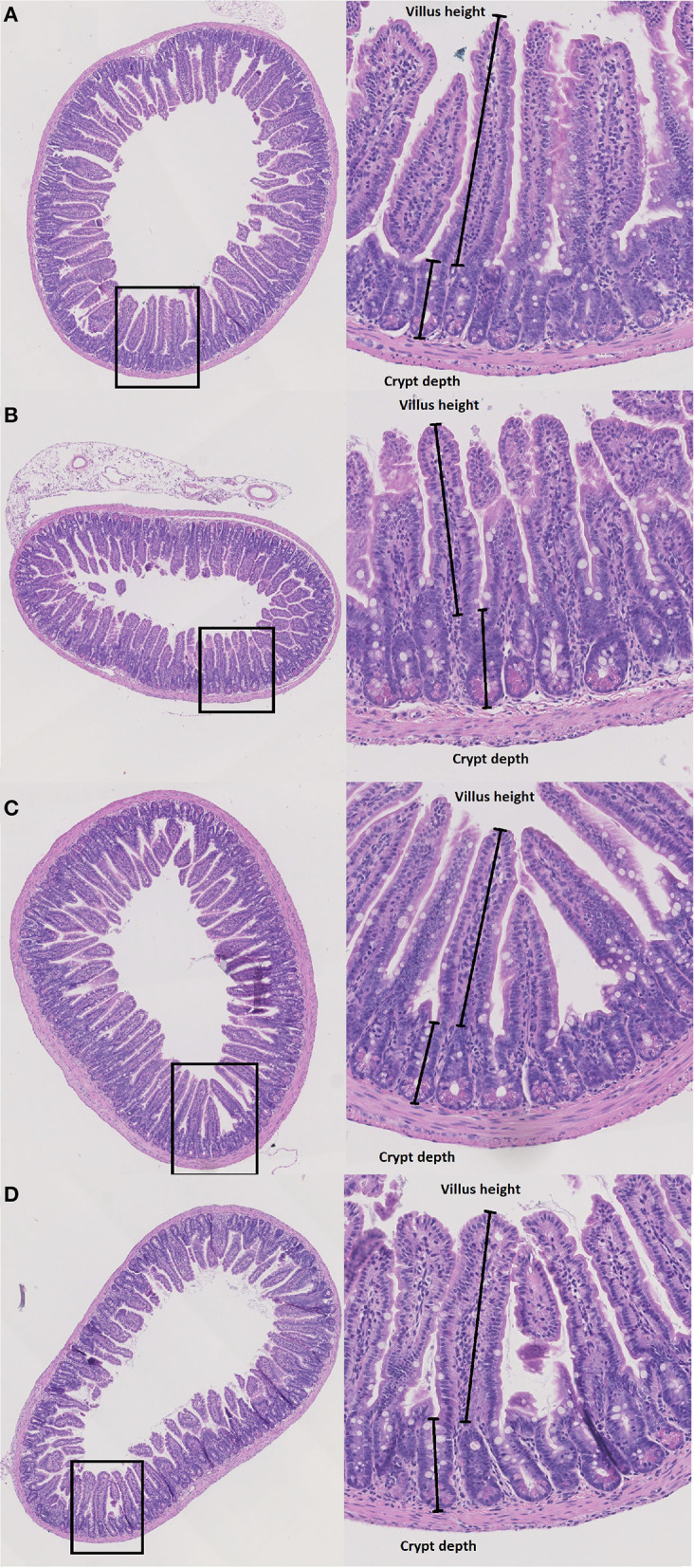
Histopathology. Villus height (μm) and crypt depth (μm) were measured at haematoxylin and eosin stained tissue from duodenum, jejunum and ileum in wildtype (WT) and knockout (KO) mice after IP injection with saline (NaCl) or 2 or 7 days after IP injection with doxorubicin (Doxo). This figure shows examples of tissue slides from KO-NaCl, jejunum **(A)**, KO-Doxo 7, ileum **(B)**, WT-NaCl, ileum **(C)**, and WT-Doxo 7, ileum **(D)**.

**Figure 4:**
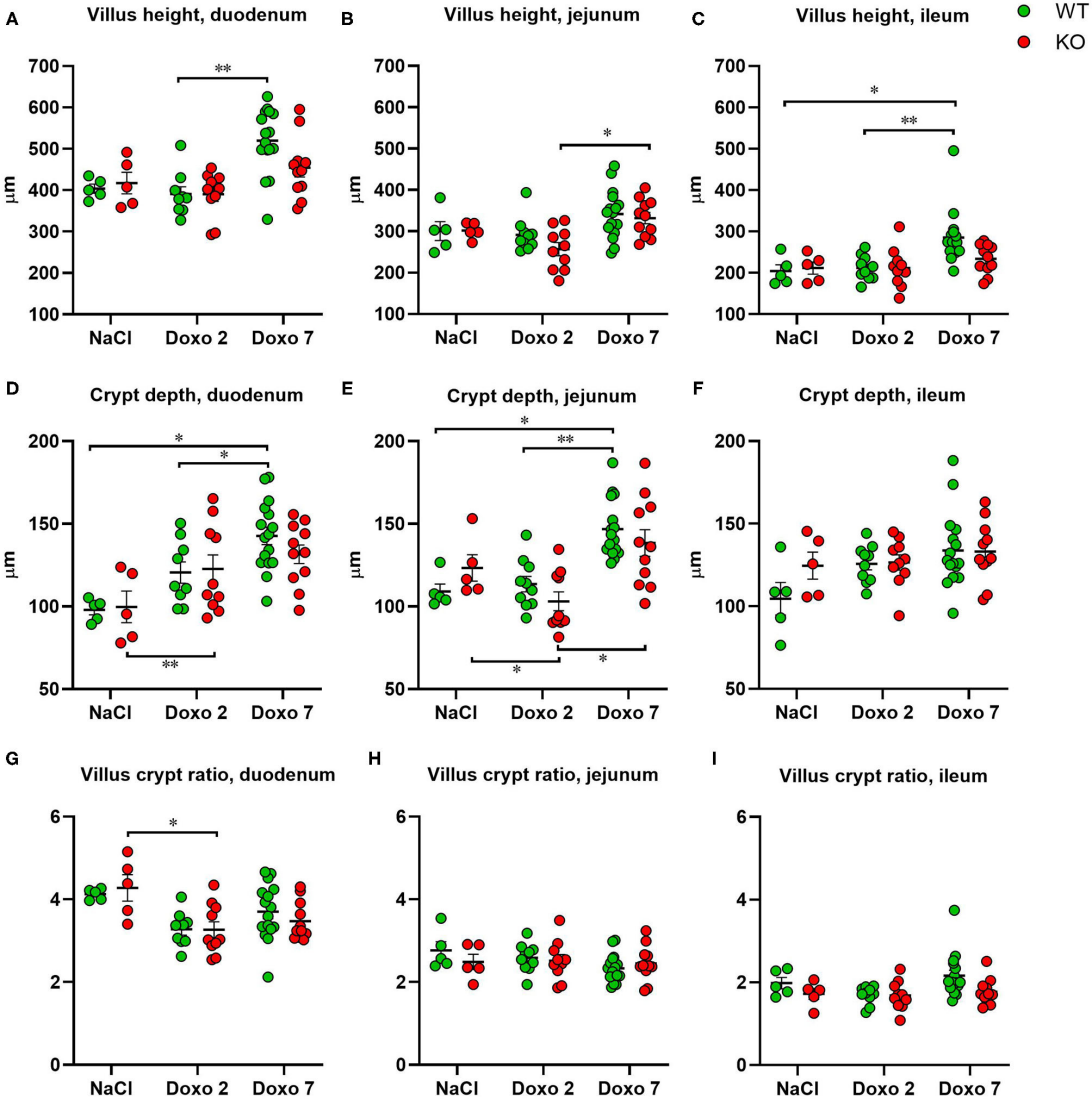
Villus height (μm), crypt depth (μm) and villus crypt ratio measured at haematoxylin and eosin stained tissue from duodenum, jejunum and ileum 7 days after IP injection with saline (NaCl) or 2 or 7 days after IP injection with doxorubicin (Doxo) in wildtype (WT) and knockout (KO) mice. Note the differences in y-axis. WT-NaCl, *n* = 5. KO-NaCl, *n* = 5. WT-Doxo 2, *n* = 10. KO-Doxo 2, *n* = 10. WT-Doxo 7, *n* = 16. KO-Doxo 7, *n* = 11. Data is presented as mean ± standard error of mean. Two-way ANOVA followed by Tukey multiple comparison test (within genotypes) and Holm-Sidak comparison test (between genotypes) were used to analyze data following a Gaussian distribution (data in **D–H**). Kruskal–Wallis test with Dunn's multiple comparison test was used to analyze data not following a Gaussian distribution (data in **A–C,I**). *p < 0.05 and **p < 0.01.

To evaluate structural damage induced by Doxo in the small intestine we measured villus height and crypt depth in HE-stained samples of the duodenum, jejunum and ileum as shown in Figures 3A–D. Doxo led to increased villus height at day 7 in the duodenum (WT-Doxo 2 vs. WT-Doxo 7, *p* < 0.01, Figure 4A), jejunum (KO-Doxo 2 vs. KO-Doxo 7, *p* < 0.05, Figure 4B), and ileum (WT-NaCl vs. WT-Doxo 7, *p* < 0.05 and WT-Doxo 2 vs. WT-Doxo 7, *p* < 0.01, Figure 4C). Doxo led to an increase in crypt depth in the duodenum (WT-NaCl vs. WT-Doxo 7, *p* < 0.05; WT-Doxo 2 vs. WT-Doxo 7, *p* < 0.05; and KO-NaCl vs. KO-Doxo 2, *p* < 0.01, Figure 4D), and jejunum (WT-NaCl vs. WT-Doxo 7, *p* < 0.05; WT-Doxo 2 vs. WT-Doxo 7, *p* < 0.01; and KO-Doxo 2 vs. KO-Doxo 7, *p* < 0.05, Figure 4E). Crypt depth was decreased in the jejunum when comparing KO-NaCl and KO-Doxo 2 (*p* < 0.05, Figure 4E). Decrease in villus crypt ratio was found in the duodenum of KO animals when comparing KO-NaCl and KO-Doxo 2 (*p* < 0.05, Figure 4G). Crypt depth in the ileum (Figure 4F) and villus crypt ratio in the jejunum and ileum (Figures 4H,I) were not influenced by chemotherapeutic treatment. There was no difference in villus height, crypt depth, or villus crypt ratio that could be attributed to the *Pglyrp2*-genotype.

### Histological Grading

**Figure 5:**
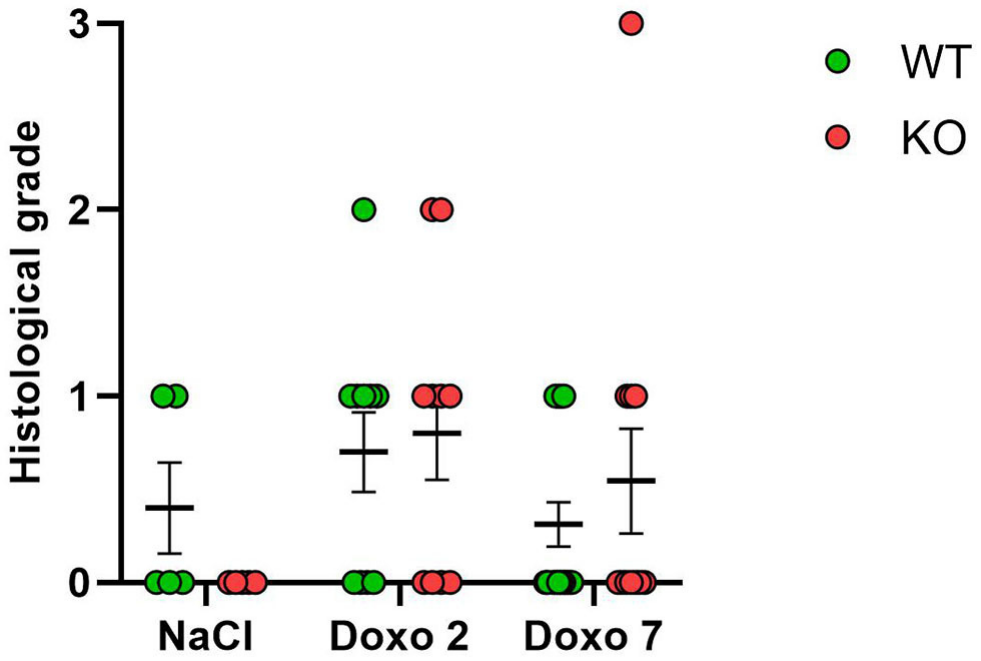
Histological score 2 days after exposure to doxorubicin (Doxo) and 7 days after exposure to saline (NaCl) or Doxo in wildtype (WT) and knockout (KO) mice. WT-NaCl, *n* = 5. KO-NaCl, *n* = 5. WT-Doxo 2, *n* = 10. KO-Doxo 2, *n* = 10. WT-Doxo 7, *n* = 16. KO-Doxo 7, *n* = 11. Data is presented as means ± standard error of the mean. Kruskal–Wallis test with Dunn's multiple comparison test was used to analyze results.

Histological grading did not differ significantly between NaCl-treated animals and Doxo-treated animals at either day 2 or day 7 after treatment with Doxo (Figure 5). No differences between *Pglyrp2*-genotypes were observed.

### mRNA Level of Inflammatory Markers

**Figure 6:**
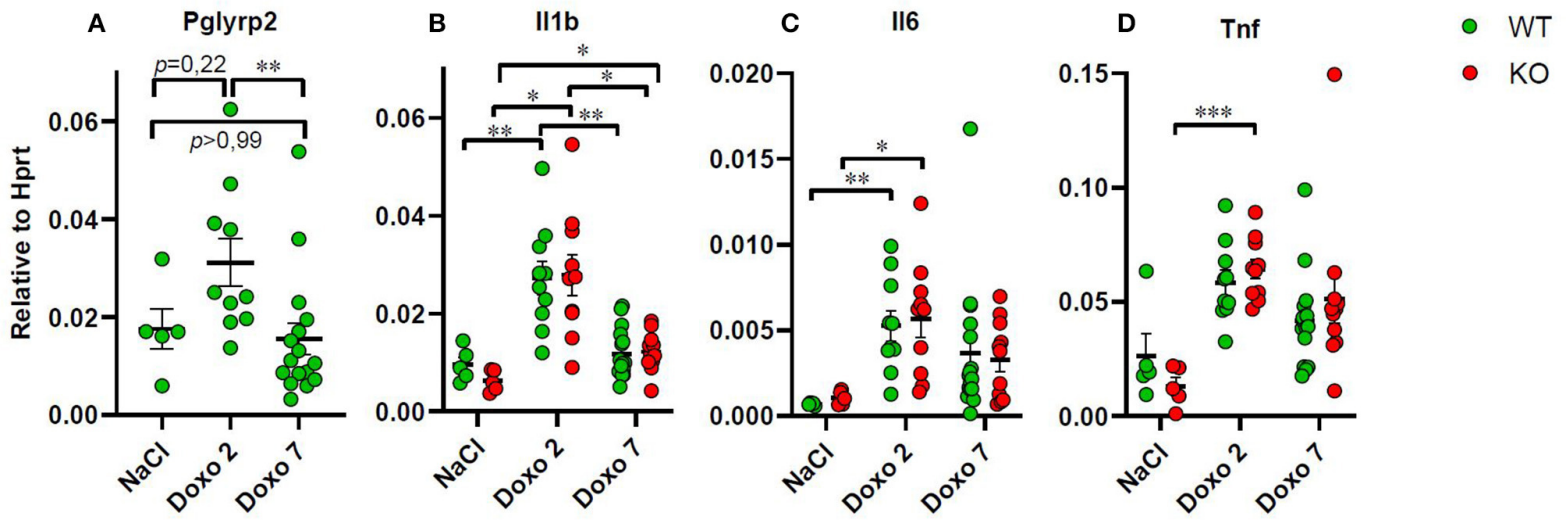
Quantitative real-time polymerase chain reaction analyses showing gene expression of *Pglyrp2, Il1b, Il6*, and *Tnf* in jejunal tissue obtained from wildtype (WT) and knockout (KO) mice either 7 days after IP injection with saline (NaCl) or 2 or 7 days after IP injection with doxorubicin (Doxo). Data are shown relative to the housekeeping gene Hprt. Please notice scale differences in y-axis. WT-NaCl, *n* = 5. KO-NaCl, *n* = 5. WT-Doxo 2, *n* = 10. KO-Doxo 2, *n* = 10. WT-Doxo 7, *n* = 16. KO-Doxo 7, *n* = 11. Data is presented as means ± standard error of the mean. Two-way ANOVA followed by Holm–Sidak multiple comparison test was used to analyze Gaussian distributed data (data in **B**). Non-Gaussian distributed data were analyzed by Kruskal–Wallis test followed by Dunn's multiple comparison test (data in **A, C, D**). *p < 0.05, **p < 0.01, and ***p < 0.001.

To evaluate how *Pglyrp2* affects the inflammatory response to Doxo treatment in the small intestine of WT and KO mice, we measured the mRNA levels of proinflammatory cytokines in jejunal tissue samples at day 7 after treatment with NaCl and at either day 2 or 7 after exposure to chemotherapy. *Pglyrp2* expression was not detected in KO animals. Doxo treatment induced the expression of *Pglyrp2* in WT animals at day 2 compared to day 7 (WT-Doxo 2 vs. WT-Doxo 7, *p* < 0.01, Figure 6A). Treatment with Doxo caused increased expression of *Il1b* (WT-NaCl vs. WT-Doxo 2, *p* < 0.01 and KO-NaCl vs. KO-Doxo 2, *p* < 0.05, Figure 6B), *Il6* (WT-NaCl vs. WT-Doxo 2, *p* < 0.01 and KO-NaCl vs. KO-Doxo 2, *p* < 0.05, Figure 6C), and *Tnf* (KO-NaCl vs. KO-Doxo 2, *p* < 0.001, Figure 6D) at day 2, which then again normalized at day 7 (for *Il1b*; WT-Doxo 2 vs. WT-Doxo 7, *p* < 0.01 and KO-Doxo 2 vs. KO-Doxo 7, *p* < 0.05, Figures 6B–D). *Pglyrp2* did not contribute to any significant differences in expression of proinflammatory cytokines on mRNA level.

### Protein Level of Inflammatory Markers

**Figure 7:**
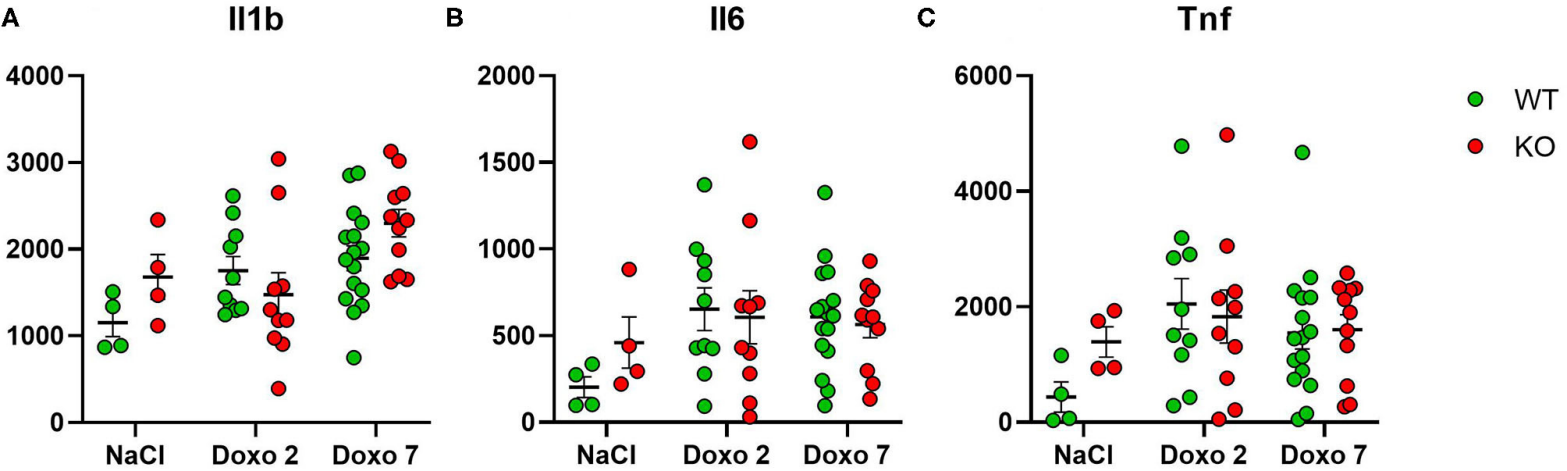
Enzyme-linked immunosorbent assay analyses showing protein expression of IL1β, IL6 and TNFα in jejunal tissue obtained from wildtype (WT) and knockout (KO) mice either 7 days after IP injection with saline (NaCl) or 2 or 7 days after IP injection with Doxorubicin (Doxo). Data are shown in pg/g tissue. Please notice scale differences in y-axis. WT-NaCl, *n* = 4. KO-NaCl, *n* = 4. WT-Doxo 2, *n* = 10. KO-Doxo 2, *n* = 9. WT-Doxo 7, *n* = 16. KO-Doxo 7, *n* = 11. Data is presented as means ± standard error of the mean. All data was Gaussian distributed and two-way ANOVA followed by Holm–Sidak multiple comparison test was used to analyze data.

To evaluate how *Pglyrp2* affects the inflammatory response to Doxo treatment in the small intestine of WT and KO mice, we measured the protein levels of proinflammatory cytokines in jejunal tissue samples at day 7 after treatment with NaCl and at either day 2 or 7 after exposure to chemotherapy. Two samples were identified as outliers by Grubbs test and therefore excluded. Doxo treatment did not cause increased expression of IL1β, IL6, or TNFα on protein level, neither when comparing protein levels within genotypes or between genotypes (Figures 7A–C). *Pglyrp2* did not contribute to any measurable difference in expression of proinflammatory cytokines on protein level.

## Discussion

In this study, we investigated a potential ameliorating effect of *Pglyrp2* in chemotherapy-induced gastrointestinal toxicity in mice. Our results showed an aggravating effect of *Pglyrp2* on weight loss. Overall, the small intestine was shortened, villus height and crypt depth were increased, and expression of inflammatory cytokines were increased after chemotherapeutic treatment on mRNA level but not on protein level, with no significant effect attributed to the *Pglyrp2*-genotype.

Doxo treated mice lost more weight compared to controls consistent with findings in similar models of CIGT in mice (26, 31, 32). We observed that *Pglyrp2* had an aggravating effect on weight loss. That finding is conflicting with the protective effect of PGYLRP2 on weight loss demonstrated in a model of dextran sulfate sodium induced experimental colitis in mice (22). The opposing effects of *Pglyrp2* on weight loss suggest that *Pglyrp2* does not have an unambiguous effect on inflammation in the small intestine and colon, respectively. Further studies including animals overexpressing PGYLRP2 may help to substantiate the current findings.

Small intestinal length and colon length have been used as surrogate markers of intestinal inflammation with shorter length indicating higher level of inflammation and vice versa in models of CIGT (33) and dextran sulfate sodium induced acute colitis (34). In the present study, small intestinal length was shortened in KO mice at day 2 and in WT mice at day 7 indicating inflammatory activity following the chemotherapeutic treatment.

Chemotherapy accelerates apoptosis in the lower regions of the crypts of Lieberkühn (35) and the height of the villi and depth of the crypts are decreased in both mice and humans at day 3 and 5 after chemotherapy (35–37). In the current study villus height was increased 7 days after administration of Doxo, and crypt depth increased 2 and 7 days after administration of Doxo. Structural damage by chemotherapy and the consequent expected decrease in villus height and crypt depth was not reflected at day 2, while day 7 in this study might have been influenced by compensatory regeneration mechanisms and this should be taken into consideration when interpreting our findings.

In another study of CIGT the histological grading of mucositis on a scale ranging from 1 to 12 showed aggravated structural damage in the mucosa 3 days after administration of Doxo compared to injection with a vehicle (36). We could not reproduce this finding after 2 or 7 days in our histological grading. This suggest that the dynamics of CIGT development may differ between different models.

Increased exposure of the gastrointestinal tract to bacteria and cytokines leads to upregulated expression of *Pglyrp2* (20), and expression of *PGLYRP2* is upregulated in jejunum of piglets after treatment with chemotherapy (25) which was also observed by RT-qPCR at day 2 in this study. Increased exposure of the gastrointestinal mucosa to peptidoglycan-coated microbes after chemotherapeutic treatment might induce the observed upregulation of *Pglyrp2* expression. Previous studies suggest that PGLYR2 is involved in the early defense against pathogens (38) and that genetic mutations in the PGLYR2-encoding gene are associated with inflammatory bowel disease (39). Two studies (38, 39) examining the effect of peptidoglycan recognition proteins (PGLYRPs) on experimentally induced murine colitis found that PGLYRPs conferred protective effects in the colon by maintaining a healthy microbiota. Increased expression of PGLYRP2 in response to chemotherapeutic treatment could occur as an attempt to maintain the normal microbiota and ameliorate the disruption of the epithelial barrier.

In the present study *Il1*β, *Il6*, and *Tnf* were induced by chemotherapy at day 2 and decreased toward baseline level at day 7 as expected (16), with no genotype differences in mRNA analysis. Contrary to the absent effect of *Pglyrp2* on inflammation in the present study, PGLYRP2 in other studies showed both anti-inflammatory and pro-inflammatory properties. Insect peptidoglycan recognition peptides have anti-inflammatory properties and protect insects from excessive inflammation by hydrolyzing pro-inflammatory peptidoglycan (20) and mutations make this protein unable to induce a protective response against Gram-positive bacteria (40). Mutation in human *PGLYRP2* abolishes its amidase activity (41) and genetic association between proteins variations in *PGLYRP2* and inflammatory bowel disease have been identified (39). In a murine model of dextran sodium sulfate induced colitis *Pglyrp2* showed anti-inflammatory properties by promoting normal gut flora and preventing induction of interferon-gamma. This anti-inflammatory effect was probably linked to the influence of *Pglyrp2* on the microbiota, because the susceptibility of *Pglyrp2*–/– mice to colitis could be transferred in stool to *Pglyrp2*+*/*+ germ-free mice (22). *Pglyrp2* also protects mice against psoriasis-like skin inflammation by promoting regulatory T cells and limiting the Th17 responses (23). In a mouse model of arthritis *Pglyrp2* KO mice were partly protected against arthritis, and WT mice had higher levels of *Il1b, Il6*, and *Tnf-*α in the foot and a higher incidence of arthritis (24). In the present study, inflammation, reflected as induction of *Il1b, Il6*, and *Tnf*, after exposure to chemotherapy was evident, but we found no phenotypic differences in inflammatory parameters when evaluating *Pglyrp2*. It is therefore likely that *Pglyrp2* plays a secondary role in the development of chemotherapy-induced gastrointestinal mucositis. Chemotherapeutic treatment may cause dysbiosis possibly associated with chemotherapy-induced mucositis, severity of enterocyte loss and systemic inflammation during chemotherapy (42–44). The effects of PGLYRP2 on the microbiota in the gastrointestinal lumen during chemotherapy remains to be evaluated.

Although doxorubicin did result in increased mRNA expression of proinflammatory cytokines, it did not affect tissue levels of evaluated cytokines. This may be a result of chemotherapy-depleted intestinal immunological cells and enterocytes producing IL6, IL1b, and TNFα as previously suggested (45). Mucositis is a highly dynamic and tissue specific process and time-dependent sequence of events, and interacting effects may explain why such mediators would in fact show an inconsistent response to cytotoxic regimens across different studies (25, 45, 46).

Due to variability in anatomy and physiology between different species our results cannot readily be translated to humans (47). That said, the current study raises clinical perspectives. We showed that *Pglyrp2* can influence weight loss caused by chemotherapy. Fifty-eight percent of cancer patients receiving chemotherapy experience weight loss and malnutrition, which increase the number of complications, adverse effects of chemotherapy and reduces quality of life (4). Therefore, it is essential to minimize weight loss in order to improve the patient's prognosis and our new knowledge in PLGYRP2 might contribute to this in the long term as we identified *Pglyrp2* as a possible predictor of weight loss.

The current study also raises further research perspectives as we found a phenotypic weight difference without determining the underlying mechanism. It seems possible that genetic influences on inflammatory response offer at least a partial explanation for the variance in patient response to antineoplastic therapy (48) and therefore further research in PGLYRP2 is relevant.

In conclusion, *Pglyrp2* aggravated chemotherapy-induced weight loss, but did not induce a specific pattern of inflammation or morphological changes in the small intestine.

## Data Availability Statement

The datasets presented in this study can be found in online repositories. The names of the repository/repositories and accession number(s) can be found at: https://figshare.com/, 10.6084/m9.figshare.13299059; https://figshare.com/, 10.6084/m9.figshare.13299032; https://figshare.com/, 10.6084/m9.figshare.13299029; https://figshare.com/, 10.6084/m9.figshare.13299026; https://figshare.com/, 10.6084/m9.figshare.13299017; https://figshare.com/, 10.6084/m9.figshare.13298999; https://figshare.com/, 10.6084/m9.figshare.14176529.

## Ethics Statement

The animal study was reviewed and approved by The Danish Animal Experiments Inspectorate.

## Author Contributions

ASB conducted the animal trial, collected data, performed laboratory analysis, made statistical analysis, and drafted the manuscript. AN contributed with everyday assistance and guidance in study setup and practical matters. AN, SH, MR, GS, AN, and UH interpreted data and thoroughly supervised during the entire process. JM and MD assisted in performing analysis in the laboratory. GM was responsible for histological gradings. AN, SH, MR, GS, MD, and JM revised the manuscript. All authors approved the submitted version of the article.

## Conflict of Interest

The authors declare that the research was conducted in the absence of any commercial or financial relationships that could be construed as a potential conflict of interest.

## Abbreviations

CIGT: chemotherapy-induced gastrointestinal toxicity
Doxo: doxorubicin
ELISA: enzyme-linked immunosorbent assay
HE: haematoxylin and eosin
IL1β: interleukin 1β
IL6: interleukin 6
IP: intraperitoneal
KO: knockout
NaCl: saline
PCR: polymerase chain reaction
PGLYRP2: peptidoglycan recognition peptide 2
TNF-α: tumor necrosis factor alpha
WT: wildtype.
