# Supplementary material for: Peptidoglycan Recognition Peptide 2 Aggravates Weight Loss in a Murine Model of Chemotherapy-Induced Gastrointestinal Toxicity
Source: Front Oncol. 2021 Mar 23;11:635005. doi: 10.3389/fonc.2021.635005 (PMC8021894; doi:10.3389/fonc.2021.635005)
Supplement: Supplementary file 1 [file Data_Sheet_1.docx]

Supplementary Material

**Supplementary Figure 1.** Overview of experimental animals. Abbreviation of the six groups are shown in bold fond. WT: Wildtype. KO: Knockout. Doxo: Doxorubicin. NaCl: Saline.

**Supplementary Table 1**. Overview of primers used in genotyping.

| *Pglyrp2*– Forward | 5’-GGCTCTCTACTCCCACACAACC-3’ |
| --- | --- |
| *Pglyrp2*- Reverse WT | 5’-GCAGCAATCCAAGCACGATCC-3’ |
| *Pglyrp2*- Reverse KO | 5’- GCCGGACACGCTGAACTTGTGG-3’ |

**Supplementary Table 2.** Overview of TaqMan Gene Expression Assays used in quantitative real-time polymerase chain reaction.

| *Pglyrp2* | (Mm01348077_m1) |
| --- | --- |
| *Il1b* | (Mm00434228_m1) |
| *Il6* | (Mm00446190_m1) |
| *Tnf* | (Mm00443258_m1) |
| *Hprt* | (Mm00446968_m1) |

**Supplementary Table 3.** Overview of assays used in enzyme-linked immunosorbent assay.

| PGLYRP2 | AMS.E15019m, Amsbio, Abingdon, U.K. |
| --- | --- |
| IL1β | DY401, R&D Systems, Inc., Minneapolis, MN, USA |
| IL6 | DY406, R&D Systems, Inc., Minneapolis, MN, USA |
| TNFα | DY410, R&D Systems, Inc., Minneapolis, MN, USA |
